# They're always there for me! Friendship and meaning in young people's lives?

**DOI:** 10.1111/sjop.12570

**Published:** 2019-09-11

**Authors:** John O'Rourke, Craig Harms, Lynne Cohen

**Affiliations:** ^1^ Edith Cowan University ‐ Mount Lawley Campus Mount Lawley Australia; ^2^ Edith Cowan University Perth Australia

**Keywords:** Meaning in life, happiness, wellbeing, secondary school

## Abstract

What gives individuals’ lives meaning is one of the bigger questions confronted by community members? Making sense of our lives and determining what it is that provides us with direction, strength, or commitment is no simple task and even more so in western consumerist societies where so many experiences appear accessible. Finding ways to elicit thoughtful responses from research participants, has led to varied approaches to this increasingly rich research area. An encouraging method is to use digital photography to extract information on what it is that captures participants’ ‘mind's eye’ when reflecting on meaning in their lives. In this article, a pilot study using a combination of digital photography and descriptive narratives was established to explore the thoughts of 174 year seven students in a private West Australian school on what provided their lives with meaning both in school and outside of school. The photos and narratives were explored for themes and while many categories were identified, it was apparent that relationships were the strongest source of meaning in their lives.

*Dedicated to Simon Bradley a man who truly understood the meaning of life*.

## Introduction

In 2009, the TV show *Australian story*, explored the story of paper plane throwing champion Dylan Parker; the episode was entitled *The meaning of life* (Chesire, 2009). Dylan had undergone brain surgery a short time before the paper plane throwing world championships, but still achieved a bronze medal. In *Australian story* he described the feelings that surrounded a perfect paper plane throw; “You just forget everything in your life, and you've got no worries and it's an amazing feeling.” Dylan felt at a young age he had discovered the *meaning of life*. Csíkszentmihályi (1990) would describe Dylan's discovery as finding flow; those things that challenge and engage you, that make you persevere at a level where skill mastery is possible, that you grow to love and that ultimately give you strength and direction. Discovering these things should make perfect sense for schools in their role of developing successful young people; surely those things that provide individuals with *meaning in their lives* would be noticed and nurtured! Of course this assumes that schools are aware of what it is that provides *meaning in life* for young people. In the pilot study reported here Steger, Shim, Rush, Brueske, Shin and Merriman's (2013) *auto‐photographical* technique was utilized (Steger, personal communication) with 174 Year 7 students, to determine what provided *meaning in life* for them both inside and outside school.

*Meaning in life* as a theoretical construct was established by the work of Victor Frankl, who, via experiences in a Nazi concentration camp, reflected on how different individuals were able to draw strength from a variety of sources and survive the harshest circumstances. Frankl (1963) maintained that humans have an instinctive desire to find meaning and significance in their lives and failure to do so causes psychological distress. His words below capture life at the very edge stripped of the niceties most citizens in the western world have become accustomed to over the last 50 years:

I told my comrades (who lay motionless although occasionally a sigh could be heard) that human life, under any circumstances never ceases to have a meaning, and that this infinite meaning of life includes suffering and dying, privation and death (Frankl, 1963, p. 104).

Frankl witnessed a life‐force that “motivates virtues and noble behaviour regardless of pragmatic circumstances” (Steger, 2014, p. 57). How Frankl's first hand observation of life at the very margins translates to today depends on the life circumstances experienced by individuals. Martela and Steger (2016, p. 531) in their thorough “conceptual refinement” break the construct of *meaning in life* into coherence (making sense of one's experiences), purpose (having a direction and future oriented goals) and significance (the worthwhileness of one's life; King, Hicks, Krull & Del Gaiso, 2006, p. 180). Thus we can see that *meaning in life* as a concept is multifaceted; nonetheless, what is common to all humans is that we have one life and making sense of this experience is central to us all.

From an adult point of view, *meaning in life* has documented associations with psychological and spiritual wellbeing (Steger, 2009), happiness (Ryff & Keyes, 1995; Steger, Oishi & Kashdan, 2009), life satisfaction (Steger, Frazier, Oishi & Kaler, 2006), and capacity to cope and adjust to stress (Thompson, Coker, Krause & Henry, 2003). Most of the academic research on this topic has explored the “amount of meaning that exists in individuals’ lives” (Steger *et al*., 2013, p. 530). Research in this area has invariably been of a quantitative nature and looked towards participants expanding their thoughts using a variety of self‐reporting scales. Crumbaugh and Maholick's (1964) *purpose in life* test and Ryff and Keyes's (1995) *psychological well‐being* scale attempt to determine whether individuals can find purpose in their lives or whether they have moved towards the “existential vacuum” (Frankl, 1967). However, the quantitative meaning in life approach, with its emphasis on rating broad categories in terms of “how much” meaning individuals had in their lives, fails to acknowledge that life meaning can be deeply personal and contextual. Given that the quantitative approach has been the predominant way in which meaning in life is studied, “what” provides *meaning in life* is at a much earlier stage of development as an area of study, particularly as it relates to adolescent students in schools. Steger *et al*. (2013) pondered this question in their research on understanding *meaning in peoples’ lives*: How does one individual person answer the question of his or her individual *meaning in life*?

The movement towards more individualized qualitative approaches to studying meaning in life was pioneered by Ebersole and colleagues who developed the *Meaning in Life Depth* approach. Throughout the 1980 and 1990s, they explored sources of *meaning in life* in several age groups – from children to elderly nursing home residents. They developed a variety of techniques to elicit student responses, appreciating that self‐reporting assessments were too limiting. De Vogler and Ebersole (1983) explored *meaning in life* for young adolescents (13–14 years of age) and asked them to rank the three most important things in their life. As was the case in their initial study with college students (De Vogler & Ebersole, 1980), these young adolescent secondary students recognized relationships, service, growth, belief, existential or hedonistic pursuits, obtaining, expression and understanding, as providing *meaning in life*. They also found three categories for *meaning in life* that were unique to the younger adolescents: activities, school and appearance.

Expanding their research base, Taylor and Ebersole (1993) explored whether a group of 26 year‐one students were able to express whether they could identify personal meaning in their lives. They were asked, “in your whole life what is most important to you?” To add age appropriateness to their methodology, the students were also asked to draw a picture of what these important things were. The students were recorded using their own writing, along with verbal responses. The authors found that the year ones they studied obtained meaning in life from relationships (42%), activities (sport or hobbies) (21%), beliefs (of a religious nature), growth (self‐improvement/meeting goals) and obtaining (receiving something nice such as a present) (8%), and school (4%). While the researchers were pleased with the capacity for these children to express thoughts on the most important things in their lives, they felt they had no capacity to appreciate how these things made them feel *fulfilled and satisfied* with life.

Extending this movement towards deeper qualitative research, Michael Steger and colleagues explored a variety of ways to generate self‐perspective on what provides individuals with *meaning in life* (Brassai, Piko & Steger, 2013; Brassai, Piko, & Steger, 2011; Steger *et al*., 2006; Steger, 2009; Steger *et al*., 2009; Steger, 2013). In recent times they have moved towards even more authentic and practical visual research methods (Steger, Shim, Barenz & Shin, 2014; Steger *et al*., 2014). This methodological shift was considered prudent, as Steger *et al*. (2013) suggest that many individuals struggle to describe experiences that are deeply personal (as in what gives *meaning in life*) both in spoken and written form. They also contend that when individuals are asked to write responses in research situations, they are in environments that provide limited visual cues (i.e., in a library, classroom, at university). Additionally, individuals may frame their responses in a manner that they imagine their audience might expect and therefore be drawn to culturally stereotyped sources. As a result of these factors Steger *et al*. (2013) used auto‐photography to elicit *meaning in life* sources from young adults. They asked 86 first year university students to take “photos of the things that made their lives feel meaningful.” They were informed that they could take either photos of real things (people, animals) or symbolic items, and did not have to represent things that were *proximal*, as they would be able to add a personal narrative to their photograph to expand their thoughts. The students took a maximum of 12 photos and then were asked to put these photos in rank order. Using an open‐coded method (Strauss & Corbin, 1990) the participant responses were categorized into a main category and then through further analysis, sub‐categories. The result of three independent reviewers and consensus meetings indicated that the most common sources of *meaning in life* were relationships, hobbies/leisure activities, nature, education and individual possessions.

Although there has been limited research in this area, *meaning in life* for adolescents can play a protective role with regard to health risk behaviours (Brassai *et al*., 2011), enhance self‐efficacy and self‐regulation (Brassai *et al*., 2013), improve career aspirations, confidence, and curiosity (Yuen & Yau, 2015) and provide young people with a coping mechanism when faced with traumatic life events (Triplett, Tedeschi, Cann, Calhoun & Reeve, 2012). For young people with complex needs, meaning in life can provide a deeper understanding of social issues and personal values (Kang, Kim, Song & Kim, 2013).

Despite this encouraging evidence of positive links between *meaning in life* and psychological health, very little is known about what provides Australian students with *meaning in their lives*. This is of concern, as the prevalence of major depressive disorders in school‐age students has increased from 2.1% to 3.2% in the new millennium (Lawrence *et al*., 2015). If this growth in mental health issues amongst our nation's young people simply reflected a downturn in economic indicators, then schools and their extended communities would clearly understand the trigger points; but these changes have occurred in a period of sustained growth in Australia, and more specifically in Western Australia during an unprecedented economic ‘boom’ (Technology and Industry Advisory Council, 2010). Perhaps this current malaise for young Australians is, as Seligman, Ernst, Gillham, Reivich and Linkins (2009, p. 293) discuss, less to do with ecological, biological or cultural factors and more to do with “modernity and perhaps what we mistakenly mistake for prosperity.” Thus, providing mechanisms and opportunities to develop and fine‐tune a deeper understanding of what is important in young peoples’ lives within the school setting, may provide tools that are critical for student's to address the fluctuations that present in their lives (Reivich & Shatte, 2003).

Thus, the current study acknowledges the growth in positive psychology interventions in Australian schools (Chodkiewicz & Boyle, 2017), and re‐works Steger *et al*.'s (2013) *minds‐eye* approach – to explore a uniquely individualized view on *meaning in life* both in and out of school, for students at a unique time in their school and personal lives. The research limited the students’ responses to four self‐selected images (Steger *et al*., 2013 encouraged a maximum of 12 ranked photos); thus creating a manageable process for potential school replication. This current study is the first of its kind to be conducted in an Australian school and will provide a deeper understanding of what it is that provides early‐adolescents *meaning in life*.

## Methodology

### Participants

The school involved in this pilot study was a large co‐educational private school of approximately 1,200 students in a relatively affluent coastal suburb of Perth, Western Australia (WA). One hundred and seventy‐four year seven students all between the age of 12 and 13 years participated in the research. There were more female participants (56%).

### Procedure

The current research was made up of four separate, but interrelated research phases.

#### *Phase 1*

The selected school had invested considerable time and resources into their positive education program: the PERMA model (Positive emotions, Engagement, Relationships, Meaning, Accomplishments) (Seligman, 2012) and were aware of the research teams previous work in the area of happiness and wellbeing (O'Rourke & Copper, 2010; O'Rourke, Cooper & Gray, 2012). After meeting with the schools Director of Positive Education and the Dean of Year Seven students, it was felt that the ‘mind's eye’ project developed by Steger *et al*. (2013) would provide a practical, student focused and potentially revealing project about what was important in young people's lives. After receiving ethics approval from the university and WA Catholic Education to conduct the research, the school principal and other key school personnel met to discuss how this research approach could connect with their existing positive education program. After these discussions, it was decided that the goals for student participation in the research would be:

1. Provide a practical method for students to focus on what provides *meaning in their lives*.
2. Provide an opportunity for students to share as a year group what *provides meaning in their lives* and to reflect on what this means to them as a community of learners, and
3. Provide an opportunity to share these types of discussions with the greater school community.

Towards the end of the third school term, the research team visited the school and spoke to the year group and school staff about *meaning in life* research, giving examples from previous studies and explaining that we were interested in *meaning of life* both *in* and *outside* school. The current research, while employing similar techniques to Steger *et al*. (2013), was quite different in its goals. While the current study represented an opportunity to reveal *meaning in life* categories for this student group, it directed the student focus onto *meaning in school life* as a specific focus as well. Additionally, the process of collecting images and providing students with opportunities to independently navigate their way through the data collection process, was not only promoted as an integral part of the research (maximizing the student's opportunities to personally reflect on their choices), but potentially as a pathway for positive education school activities. The process of taking two photos about *meaning in life in school* and two about *meaning in life outside of school* was discussed with the students, and how they would send these to a school wiki created for the research. The nature of the photos was clarified, in that they could be about people, animals, places, beliefs, and possessions as well as visual representations of relationships and feeling. As with Steger *et al*. (2013), it was discussed that the photos could be derivative of their *meaning in life* choice, as it could be explained via their personal narrative.

#### *Phase 2*

Consent forms were sent home to parents (*N *=* *238), and of these, 186 parents agreed for their children to participate in the research. Over the first 2 weeks of the last term of the school year (term four in WA), students took images using commonly available technology (e.g., mobile phones or tablets) and sent through these images to the wiki established for the study. A research assistant printed the digital images into color hard copies and, using their pastoral care group (PCG) names, devised a code that identified the student without naming them. The images were then collated, the identifying code for each individual written on the back of each hard copy (a separate code existed for in school and outside of school images), and then organized the images into the students’ PCGs.

#### *Phase 3*

Upon organizing the hard‐copy digital images into eight separate PCG folders, the research assistant and chief researcher met the students in their PCGs over a week (there were 8 PCGs – mean number of students in each was 30). During the PCG meetings (which took approximately 40 minutes), the students collected their hard‐copy digital images and were first asked to write a brief narrative about *why* the two digital images they chose gave their *school lives meaning*. They were asked to do this quietly and respectfully and were reminded to give “special thought” to the narratives. Next, the students were asked to write a brief narrative about *why* the other two digital images they chose gave their *out of school lives meaning*. Students were asked to check that they had completed all narratives before the narratives were collected by the research assistant. On average, the girls wrote 37 words per image, while the boys wrote 25 words per narrative. To complete this data collection phase, the students completed a self‐reported happiness scale. Due to absentees and timetable clashes, of the 186 students that had consented to participate and had submitted photos, 174 students completed this phase of the research.

#### *Phase 4*

The hard copy images with their associated narratives and consent forms were photocopied by the research team and the data was entered into EXCEL spread sheets by a research assistant. The original color hard copies of the images were returned to the students in a follow up PCG class, and the variety of *meaning in life* sources were discussed. Students were encouraged (where they felt comfortable) to volunteer their reasons for selecting these sources, and the PCG teacher coordinated these choices and looked for similarities, differences and patterns. The students then typed up their individual narratives associated with the images and printed these thoughts so they could be displayed with their A4 size photos. Those students that consented to display their photos and narratives resubmitted them to school staff (n = 114 – 64% of participants), who then placed the images on partition boards and displayed them (along with information on the research project) in the foyer of the school hall for a 1 week period. Parents were encouraged to visit this display and, given that the display of the images occurred towards the end of the year (a busy time for the school), a large number of school community members viewed the installation. It was felt that this installation highlighted aspects of positive education, and demonstrated that the school valued the thoughts, interest and aspirations of their youngest community members.

### Data analysis

Utilizing the analytic inductive coding approach employed by Steger *et al*. (2013), the first author and a trained research assistant (a Master's of Psychology student) established initial categories for *meaning in life in school* and *meaning in life outside of school* based on the face‐value of images selected by the students. Consistent with Steger *et al*. (2013, p. 535), the researchers were not trying to develop a theory but were rather attempting to establish “representative categories of sources of meaning.” Subsequently, only *open* coding (establishing categories from the initial images) and *axial* coding (examining categorical intersections) were conducted.

In the open coding phase of the data analysis, the two coders independently examined each student image and narrative to establish the initial source of meaning for the image. These initial codings were organized into a main category and sub‐category. For example, it was not uncommon for students to select an image that on initial viewing was categorized as “holidays,” however, the narratives revealed that the selection was more about time spent with family. In some instances, images were sorted into multiple categories. After the completion of the independent coding, the two researchers reviewed the initial categorization of their research colleague (initial percentage agreement for *meaning in life* sources in‐school was 80% and out of school 75%). Over a period of several hours, more fine‐grained consensus on categories and sub‐categories was achieved. As with Steger *et al*. (2013), the process was extended so that images not strictly aligned to categories and beyond the parameters of connected sub‐categories were given new more specific categories to represent meaning. This process was continued until all images were categorized and the researchers could no longer find images that did not have a clearly defined category.

## Results and Discussion

**Figure 1:**
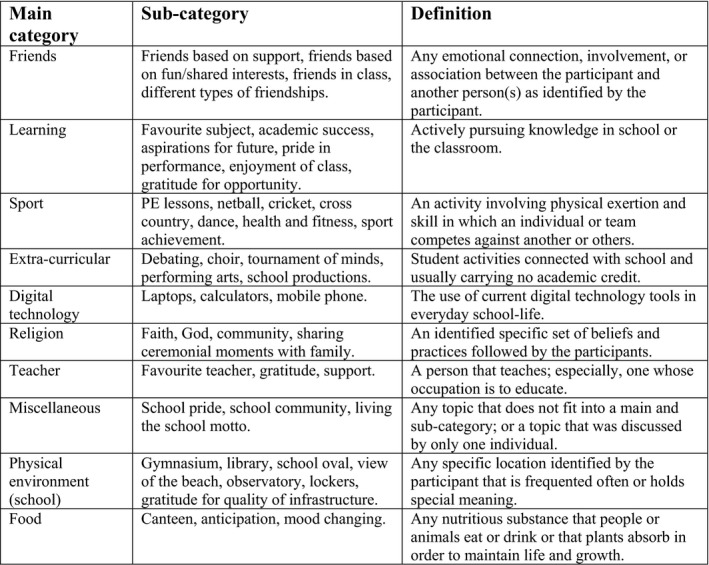
*Meaning in school life*: main categories and subcategories in participant's photographs.

**Figure 2:**
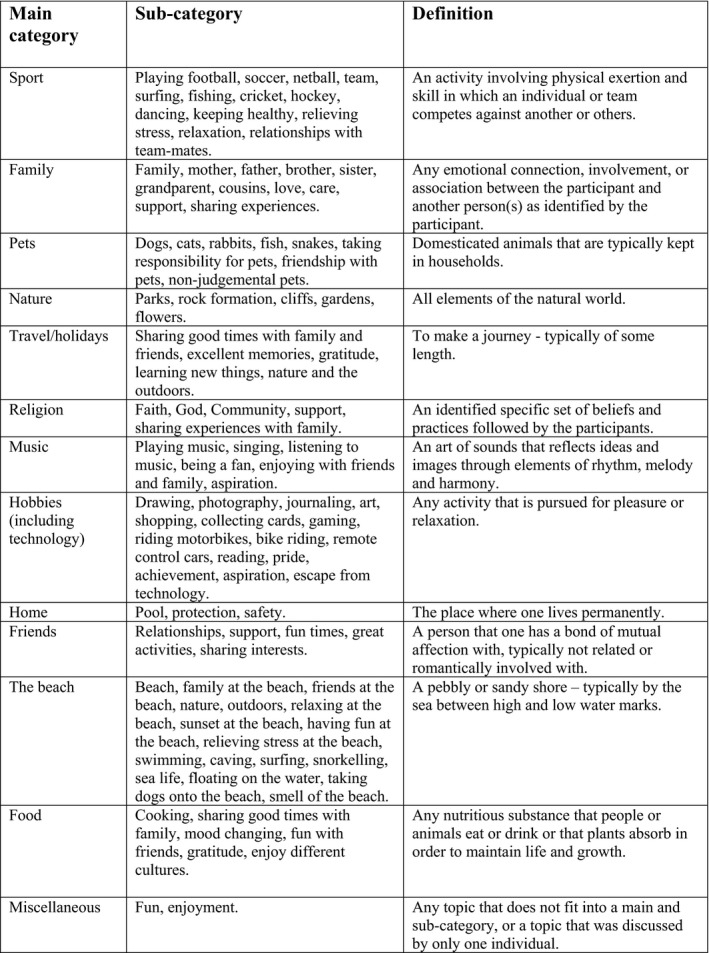
Meaning in life outside school: main categories and sub‐categories in participant's photographs.

The student's main categories for *meaning in life in school* and *meaning in life outside school* were established and are shown in Figs. 1 and 2.

### In school meaning in life

The students established a variety of different categories that provided their *school* and *outside of school lives* with meaning. In terms of what gave their *school life meaning*, eleven sources were identified: (1) friendships, (2) learning, (3) sport, (4) school physical environment, (5) extra‐curricular, (6) digital technology, (7) teachers, (8) religion, (9) food, (10) self, and (11) miscellaneous. As with previous research exploring *meaning in life* in educational settings (De Vogler & Ebersole, 1980; De Vogler & Ebersole, 1983; Steger *et al*., 2013; Taylor & Ebersole, 1993), a high percentage of sources (82% – see Table 1) attributed *meaning in life* to friendships, activities, and learning.

**Table 1:** *Frequency with which participants took photographs within each category of* meaning of life *inside school*

Main category	Number (%) of participants submitting matching photograph theme
Friends	144 (42%)
Learning/Education	102 (29%)
Physical environment	36 (10%)
Sport	29 (8%)
Extra‐curricular	10 (3%)
Miscellaneous	7 (2%)
Religion	6 (2%)
Digital technology	5 (1.5%)
Teachers	3 (0.5%)
Food	2 (< 0.5%)
Family	1 (< 0.5%)
Self	1 (<0.5%)

As a result of the *photo‐elicitation* approach, these sources were broken up further into sub‐categories after analysis of their narratives (see Fig. 1). In comparison to Steger *et al*. (2013), exploration of *meaning in life* for college students, the categories were less varied and more obvious in their sources (whereas the college students more often used images such as their cars to represent freedom, status, friendship, etc.). This finding was possibly explained by the students in Steger *et al*. (2013) taking up to 12 photos, thus identifying a wider variety of sources often reflecting life experiences the younger students in this current research have had little exposure to (i.e., traveling independently with friends, being financially self‐reliant, being in a romantic relationship, etc.). Nonetheless, the unique aspect of this research is that it allowed participants to break down their sources of *meaning in life* into finer detail. For example, learning/education (De Vogler & Ebersole, 1980; De Vogler & Ebersole, 1983; Taylor & Ebersole, 1993), was dissected to include love of subject, aspirational learning and how learning was connected with their futures, the joy of academic success, the gratitude of being given an opportunity to receive a good education and the difference learning with friends makes. Furthermore, friendships that were revealed to provide the most *meaning in school life,* again could be broken down into different types of relationships based on enjoyment of a subject or activity, shared interests and the capacity of friends to offer support. Interestingly, students in the present study rarely highlighted classroom teachers as being a source of *meaning in school life* and often revealed in their narratives that friends provided them with support both academically and emotionally.

**Figure 3:**
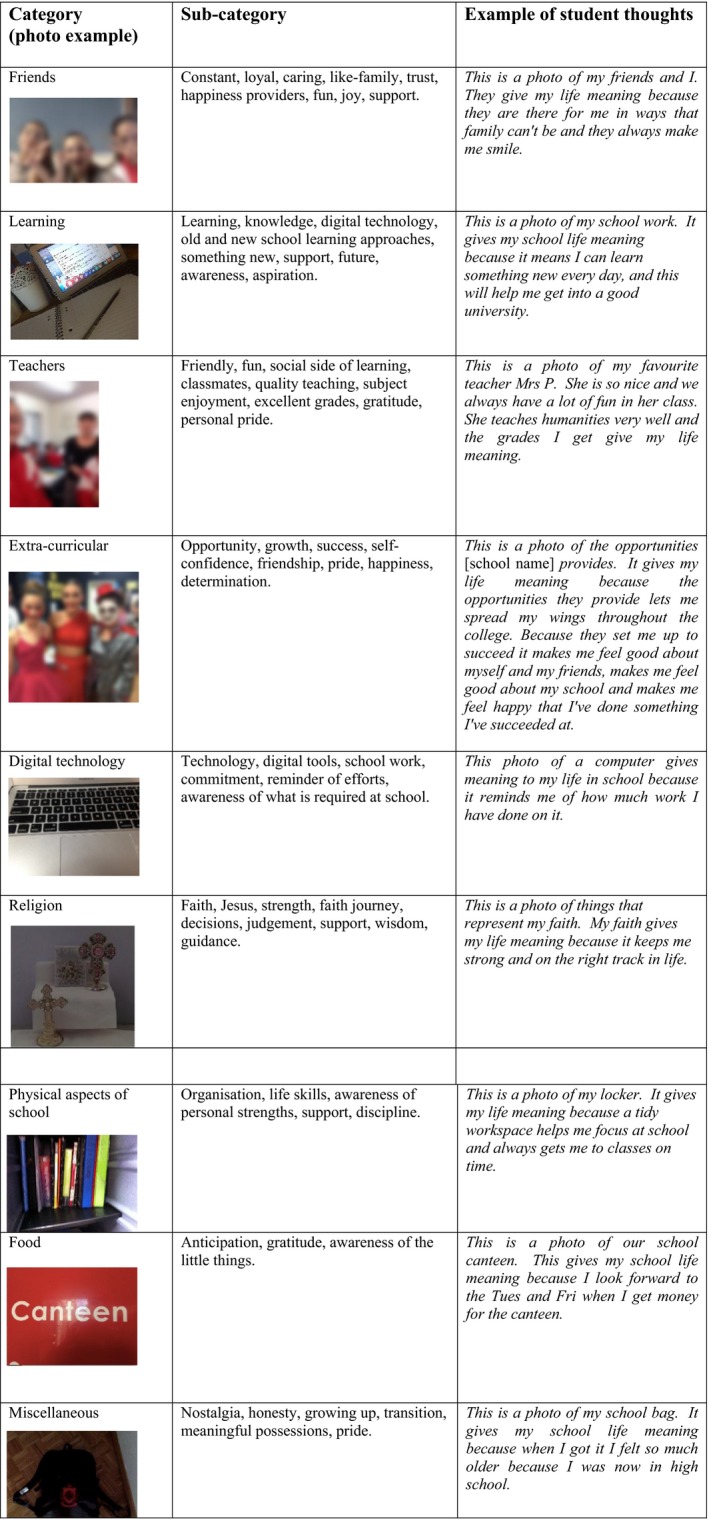
*Meaning in school life*: main categories and sub‐categories in participant's photographs. [Colour figure can be viewed at http://wileyonlinelibrary.com]

Initial analysis of images selected by the students, showed students unpacking common categories of *meaning in school life*, only to reveal subtler references to loyalty, trust, inspiration, care, love, and gratitude. For example, Fig. 3, a student represents learning via a file and a computer screen, but reflects in the narrative that it symbolizes the joy of learning and the mechanism for aspirational pathways. Additionally, a student points out that her extra‐curricular activities such as the school production are a source of *meaning* in her school life*,* but additionally reveals there are multiple reasons for this including, appreciation of the opportunity, personal growth, feelings of success, self‐confidence, friendship, personal pride, school pride, happiness, and determination. Thus, as with Steger *et al*. (2013, p. 535), the *auto‐photographical/photo elicitation* revealed familiar sources of *meaning in life,* but also revealed ‘unique paths’ to these well‐known themes.

### Out of school meaning in life

**Figure 4:**
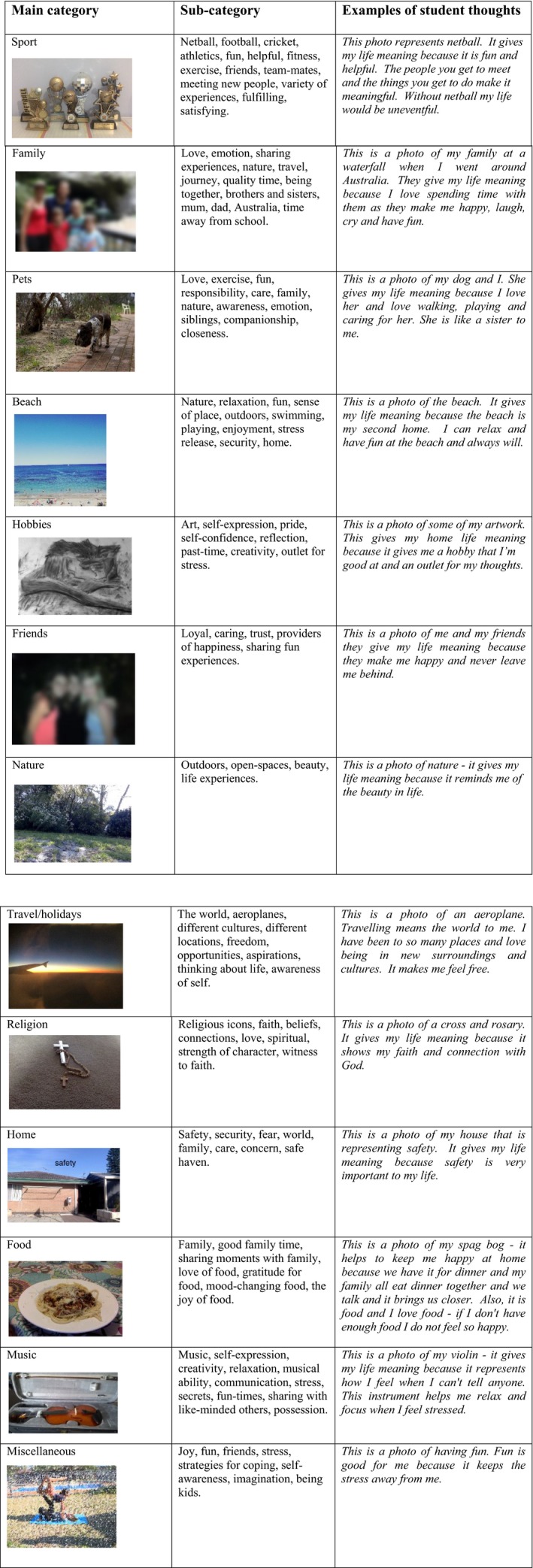
*Outside of school*: main categories and sub‐categories in participant's photographs. [Colour figure can be viewed at http://wileyonlinelibrary.com]

In their exploration of *meaning in life out of school*, again students gravitated towards relationships mainly with family, friends, and pets (Fig. 4). Twelve sources of meaning were revealed: (1) sport, (2) family, (3), pets, (4) friends, (5) nature (6) the beach, (7) travel, (8) religion, (9) hobbies, (10) music, (11) food, and (12) miscellaneous. As revealed in previous research when exploring *meaning in life* (De Vogler & Ebersole, 1980; De Vogler & Ebersole, 1983; Steger *et al*., 2013; Taylor & Ebersole, 1993), relationships, activities, and nature were all important sources. While activities have been identified previously as important sources of meaning, in this present study it was the biggest source of *meaning in life*. Again, the photo‐elicitation process allowed sport/activities to be explored in more detail. Thus, while the love of specific sports was important (such as netball, cricket, soccer, Australian rules football, athletics), *meaning in life* was often attributed to; being part of a team; spending time with friends; providing an aspirational goal; and relieving stress and pressure. For example, the student comments below point to a love of the sport, but it provides *meaning* because it is shared with others:

- *This is a photo of swimming comps. It gives my life meaning because it's really fun to do and watch. It is also a really great sport to cheer on your friends in the stands*.
- *This is a photo of my footy [sic Australian Football]. It gives my life meaning because by playing footy it allows me to catch up with my mates and play the game we love*.

The sources of *meaning in life outside school,* had similarities with the *in‐school meaning in life*, in that the important things involved relationships and activities, such as sport, hobbies, and travel; but also localized sources were revealed, for example, a source of *meaning in life* quite often identified (8%) was the beach. Initially, this category was placed under nature, but given the number of students who were specific about the beach and why it was important (e.g., having fun, relaxing, stress‐relief, quality time with family, exercise, activities like surfing, snorkeling, swimming and even described by one student as *my second home*); it was determined that this source was a distinct category Table 2.

**Table 2:** *Frequency with which participants took photographs within each* meaning of life *category outside of school*

Main category	Number (%) of participants submitting matching photograph
Sport	104 (30%)
Family	88 (25%)
Pets	51 (14%)
Beach	28 (8%)
Hobbies	25 (7%)
Friends	18 (5%)
Music	13 (4%)
Travel	10 (3.5%)
Nature	6 (2%)
Food	6 (2%)
Religion	6 (2%)
Home	3 (1%)
Miscellaneous	1 (<0.5%)

While the coding method explored categories and sub‐categories, the thorough examination of the image and narrative, revealed that strong themes emerged from many of the student selections. For example, while many students chose sport, activities, extra‐curricular commitments, travel, the beach, or nature as something that provides their *in‐school* and *outside school lives* with meaning, quite often they identified that it was meaningful as they were able to share this experience with friends or family. As such, many of the images and narratives selected were even more focused on relationships than the initial categorization. Seventy percent of all images selected by students for *outside school* meaning, and 62% *in‐school* meaning had direct references to the importance of relationships. Further, within these categories 39% of students *outside school* and 44% *in‐school*, pointed out that the image selected provided them with support and a coping mechanism.

While it is difficult to analyze the inherent meaning of narratives on *meaning in life* sources, what became evident was that relationships with friends were deeper than simply providing good company or having fun together. While fun and the enjoyment of each other's company were evident, the narratives often described relationships that were built on trust, loyalty, support, and *being there* for each other. Seventy‐six percent of student narratives described their friends as being supportive or positively influencing school. Thirty‐one of the 151 images, focused on friendships in school used the expression “they are always there for me.” While this may simply be a turn of phrase, the notion that their friends are “always there for them” may point towards friendships based on deep commitment and emotional support as well. That these students see these friendships as such a strong source of *meaning in life* and that these relationships appear to be “intense” even at the early stages of secondary, provide schools with much to reflect on during these student's school journey.

To explore *meaning in life* sources in more depth, each narrative was analyzed and categorized into the three facets theorized by Martela and Steger (2016): Coherence, purpose and significance. Martela and Steger (2016) acknowledge that sources of meaning may be different to the actual experience of meaning but nonetheless contribute to ones sense of meaning. Thus, the sources presented may not directly capture these young people's emerging sense of *meaning in life*, but allude to what it is that shapes their views.

Table 3 identifies that for the majority of students (75%) *meaning in life* sources were focused on *coherence* that is, examining life through a lens of understanding. Sources that identified a clear sense of *purpose* for this group were considerably less obvious (23%), and further identifying a source of *significance* that “lifted life beyond the normal” was even less apparent (3%). Slight variations became apparent when exploring the facets in *meaning in life* for this student group from a *school* or *out‐of‐school* point of view. These variations occurred around *purpose*, whereby *in‐school* narratives made up 28% of *meaning in life* sources, whereas for *out of school* this was only 17%. This is not surprising as school is often seen as the vehicle by which young people develop a sense of their own personal calling (Graham, Van Bergen & Sweller, 2015). However, for this student group most *meaning in life* sources were still focused on developing understanding of their experience (generally via relationships), rather than deriving clarity around the specific purpose of school. Finally, students rarely attached *significance* to events via a deeper sense of what the *meaning in life* source meant to them. Those that did, focused mainly on holidays and how important these were to them and their families, but some individuals were able to appreciate both their school and outside life at a deeper level than other peers, for example:

**Table 3:** *Student* meaning in life *sources categorized by Martela and Steger's (* *2016* *) three facets of* meaning in life

	School meaning in life	Out of school meaning in life	Total
Coherence	234	248	482
Purpose	95	54	149
Significance	7	11	18
Total	336	313	639

This is a photo of my laptop and my work. It brings meaning to my life because it reminds me of how hard my mum and dad work to get me a good education.

### Self‐reported happiness

The participating students also rated their happiness using a self‐reported faces scale. The faces scale was originally used by Andrews and Withey (1974, 1976) and was seen as a simple but robust scale with a validity coefficient of 0.7–0.82. The scale has been used in previous happiness research (O'Rourke & Copper, 2010; O'Rourke, Cooper & Gray, 2012; Holder & Coleman, 2008) and is ideally suited to working with primary/early secondary students because it does not rely on the reading comprehension of participants.

Pleasingly, at least 92% of all student responses in this study were within the three happiest categories (see Table 4). These results are similar to O'Rourke and Copper (2010) Australian survey of year 4 and 6 students and Holder and Coleman's (2008) study of Canadian students aged 9–12 years. In the present study, the happiness ratings of boys (5.58/7) and girls (5.6/7) were similar.

**Table 4:** *Percentage of respondents within each category of the Faces Scale*

Score							
Respondent Group	1	2	3	4	5	6	7
	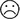	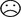	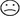	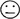	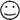	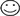	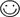
Happiness (F)	0.0	0.0	0.0	9.2	34.2	44.7	11.8
Happiness (M)	0.3	0.0	0.0	7.1	32.6	52.0	8.1
Happiness (Tot)	0.0	0.0	0.0	8.0	33.3	48.8	9.8

To explore relationships between self‐reported happiness and *meaning in life* source, the researchers entered each individual student's category of *meaning in life* (i.e., friends, sport, religion) into a SPSS spreadsheet to determine if any sources explained higher levels of happiness. Further, the student's self‐reported happiness was compared with their choice on whether they would display their photos and narratives in a school installation for public viewing.

The spread of sources of *in‐school meaning in life* were relatively even, and regardless of self‐rated happiness “friends” and “learning” was most often identified. There was a slight tendency for happier students (Level 6 and 7) to have nominated a greater variety of *meaning in life* sources, but a larger sample size would be necessary to determine if there was any significance to this observation. As with the *meaning in life* sources in school, the *out‐of‐school meaning in life* sources were distributed in a uniform manner. Sport and family were clearly the *meaning in life* sources most identified by students regardless of self‐reported happiness levels. As with in‐school *meaning in life* sources there was a suggestion that the happier students (Level 6 and 7) identified a greater variety of *meaning in life* sources, but again this is only speculative without a larger sample. What was noticeable for the students in the lower range of happiness (Level 4 and 5) was a proportionally higher frequency of pets as a *meaning in life* source than those in the happier range within their year group. The numbers are again too small to speculate on why this group of students may find this *meaning in life* source so important; however, pet/human relationships tend to be safer and easier to manage than typical human interactions (Nebbe, 2001). There was no statistical relationship between self‐reported happiness and whether the individuals made the choice of displaying their photos and narratives to the wider school community.

## Overall discussion

As Steger *et al*. (2013, p. 536) discuss, *meaning in life* appears to be currently “enjoying a burst of energy and excitement.” While historically research in this area has seen the implementation of psychometrically robust questionnaires and surveys, qualitative approaches such as the *minds‐eye* begin to reveal varied sources and multi‐layered thought towards *meaning in life*. Burrell (2017, p. 30) discusses the importance of ‘something to live for’ and connects awareness on *meaning in life* with ‘purpose’. It may seem fanciful to imagine that young adolescents have a clearly defined purpose in their lives, but the research method employed in the present study allowed these students to provide deeply personal and reflective material that revealed clarity around what provided *meaning in their lives* and why this was the case. Thus, what this study reveals for the first time is what sources are important in these young adolescents’ lives using an innovative and inclusive approach. The students presented deep and sometimes surprising thought around how their *meaning in life* sources influenced their young lives.

For the adolescent participants in the current research, *friends* and *learning* appeared critical in providing *meaning in life in‐school*. *Outside of school*,* sport* and *family* were identified as providing the most *meaning in life*. While these findings align with previous research, the *minds‐eye* approach provided the opportunity to break down these categories into individualized accounts of *meaning in life*, for example, school friendships built around loyalty, trust, support or shared interests. While Steger *et al*. (2013) identified relationships amongst college students as being similarly important, a focus on values and possessions was more evident in their study and perhaps represented markers of life maturation and world weariness thankfully not evident for the students in this current study.

So what do the findings from the present study reveal about the young people whom agreed to participate in the present study? The students reflected earnestly on what sustains their lives in their first year of secondary school and this communicated an emerging view on personal independence and awareness that support in a variety of forms was vital. More than this, it revealed that many were able to articulate the school learning journey they were on via several sources, namely, the enjoyment of a subject or specific class, a desire for academic success, aspirations for the future, general pride in performance, the enjoyment of being in a specific class, and gratitude for the opportunity that school presented. Further, the students revealed that their friendships both in and outside of school even 1 year post primary school were perhaps deeper than educators imagine. While family provided many with a great sense of *meaning in life* (invariably these students’ narratives were built around love and shared experiences), when describing friends, they used expressions such as being constant, loyal, caring, family‐like, trustworthy, happiness providers, joyful, and supportive. Students consistently revealed that their friends were mentors and guides. For example, one participant stated: “this is a photo of my friends, they give my life meaning because they are always there to help me and inspire me to do great things. They are positive people who don't bring me down ‐ they make me feel better about myself”. As to whether this strength of connection between friends is deeper now than at other times as a consequence of online activity outside of school is conjecture, but it becoming clearer that the internet plays a critical role in the formation of close relationships for young people (Borca, Bina, Keller, Gilbert & Begottit, 2015; Lenhart, Purcell, Smith & Zickuhr, 2010).

Martela and Steger's (2016) theorizing around the three facets of *meaning in life*: coherence, purpose and significance, presented an interesting way to examine the student *meaning in life* sources and narratives both in and outside school. Invariably, students *meaning in life* sources pointed towards developing *coherence* of their life via a love of sport, music, drama, travel, books, the beach and more often their strong connections with friends and family. A belief for these young people that their sources of *meaning in life* made sense of their sometimes‐confusing worlds was apparent, for example, “This is a photo of me and a friend [sic]. It gives my school life meaning because friends make me happy which is a huge help when you are in a tough time.”

Additionally, this present research established on a small scale that a clear sense of purpose was emerging for some students. While still being predominately focused on making sense of their lives, some students had clarity around what provides *purpose* in their lives even in early adolescence. For example, in this instance a student reflects on a national gymnastics title and draws upon why this provides her with *meaning in life*: “It's a life commitment to me and to be completely honest my life so far has revolved around it.” In recent research that explored the connections between life events (identified as the singular most personally meaningful event of the last fortnight) and *meaning in life*, Martela, Ryan and Steger (2018) explored factors that are aligned to life significance and purpose. They found that specific needs satisfaction and positive affect associated with the event explained a significant part of variance for purpose in life (70%) and significance in life (60%). Thus, it is worth speculating that some of the positive narratives described by the students in this research may be important in developing longer‐term meaning in their lives.

What also emerged was that *purpose* appeared to be more associated with *school* than *out of school*. Hence, schools should appreciate while most students are looking to make sense of their lives, some (even in their first year of secondary) appear to have clarity around a bigger picture and are beginning to be driven by strong motivators. It would be worthwhile comparing the student's *meaning in life* source narratives with the college students in Steger *et al*.'s (2013) *minds‐eye* research. Although the *meaning in life* sources were similar in Steger *et al*. (2013), as they were college students it would be understandable that their narratives reflected a clearer sense of *life purpose*.

Students within this research also completed a self‐reported happiness faces scale to determine if there was any connection between *meaning in life* sources and overall happiness. There was no statistical evidence to suggest that students who selected specific *meaning in life* sources were happier than others. Nonetheless, given 90% of the participating students self‐selected that they were happy most of the time and most in‐turn chose relationships in one form or another as a *meaning in life* source, is indicative of the influence of friends, team‐mates and family towards wellbeing (Holder & Coleman, 2008; O'Rourke & Copper, 2010).

### Limitations and future research

Some limitations with respect to this current research are identified. This study draws on a limited pool of participants and as such represents a snapshot of a group of same‐aged individuals from similar SES backgrounds. The school population was from a reasonably homogenous ethnic background, and therefore what provides *meaning in life* to these individuals may not reflect the growing diversity of the Western Australian metro and regional population. Further, the schools’ close proximity to the beach, student access to quality infrastructure (such as a new gymnasium, library, performing arts center) and the school's commitment to pastoral care and positive education, identify that these factors may have impacted on the selection of the student's *meaning in life* categories (Prager, Savaya & Bar‐Tur, 2000).

In terms of *meaning in life* sources, the choices made by students at school may have impacted on those identified outside of school, although no strong patterns emerged. There were examples of students selecting friends in both environments, but more often students identified friends in school, and sport and family outside school as providing *meaning in life*. As discussed, many students selected sport as an outlet to connect with friends, rather than the love of the sport itself. It could be argued that these individuals were selecting friends both *in‐school* and *out‐of‐school* as providing *meaning in life* further highlighting their importance.

Finally, research examining similar schools in regional or single‐sex settings; those in lower SES metro locations, or regional and remote schools may have provided a greater diversity of *meaning in life* sources. Additionally, schools in a variety of international locations; schools that do not run positive education programs, and of course researching students from different age groups, would add to these current findings and provide a deeper contextualized awareness of *meaning in life* in and outside of the school environment.

While acknowledging these limitations it is important to recognize that this current research provides the first glimpse of the sources of *meaning in life* for young Australians *in* and *outside of school*. The methodology used in the present study was aligned to the earlier methods of Steger *et al*. (2013, p. 537), and as such provides further evidence towards an awareness of new variations on larger themes in life meaning that are “bottomless yet meaningful.” The rich data provided by the narratives points towards Schnell's (2014) discussion that there is a fourth dimension of *meaning in life*, namely; being a part of something bigger. While Martela and Steger (2016, p. 541) suggest Schnell's assertion around *belonging* might be better understood as a strong source of source of meaning rather than a “facet of meaning,” it was not difficult to see how being connected whether through friendship, sporting clubs, families and natural elements was a constant thread of thought that emerged from this research.

## Conclusion

There are three directional themes emerging from this current study. First, as with Steger *et al*. (2013) what may emerge from research such as this is that secondary students, via practical interventions (such as the *minds‐eye* process) and accompanying teacher‐led discussions*,* can develop a greater appreciation of what provides them with *meaning in life*. This greater appreciation of personal meaning may allow them to reflect on and appreciate what is sustaining them at critical points in their lives and potentially provide added resilience to the challenges that life inevitably presents (Martela & Steger, 2016; Thompson *et al*., 2003). That the self‐selected student images in this research were used to create an installation on *meaning in life* for the wider school community, further reveals the ways schools can reinforce a commitment to positive education and improved mental health. Second, while students at early secondary are still developing their understanding of the world they live in, *in‐school* and *outside‐of‐school* experiences beginning to establish a clearer sense of purpose in their lives. Thirdly, practical research undertakings such as this may provide schools with a deeper awareness of what is significant to their students throughout their secondary years and what it is that makes their “life worth living” (Martela & Steger, 2016, p. 541). In appreciating their students more acutely, schools can tailor intervention programs, and enhance existing mental health strategies more accurately.

The current research provides further evidence of the variety of sources of *meaning in life* for young people, and reinforces the awareness that friends, family, team‐mates and relationships in general are at the core of their existence. As with all research that provides a voice for young people, the challenge for schools and society are to appreciate its genuineness and respond in kind to its message. All of us as parents, relatives, educators, coaches, and friends can appreciate this simple, but powerful message from one of the students in the research:

This is a photo of my friends – they give my school life meaning because they care for me, they are a lot of fun to be around, they make sure I'm OK.
